# Ceftazidime/avibactam versus polymyxin B in carbapenem-resistant *Klebsiella pneumoniae* infections: a propensity score-matched multicenter real-world study

**DOI:** 10.1007/s15010-024-02324-8

**Published:** 2024-06-17

**Authors:** Hai-Hui Zhuang, Qiang Qu, Wen-Ming Long, Qin Hu, Xiao-Li Wu, Ying Chen, Qing Wan, Tian-Tian Xu, Yue Luo, Hai-Yan Yuan, Qiong Lu, Jian Qu

**Affiliations:** 1 Department of Pharmacy, the Second Xiangya Hospital, Institute of Clinical Pharmacy, Central South University, Central South University, No.139 Middle Renmin Road, Changsha, 410011 China; 2 Department of Pharmacy, Xiangya Hospital, Central South University, Changsha, 410078 China; 3 National Clinical Research Center for Geriatric Disorders, Xiangya Hospital, Central South University, Changsha, 410078 China; 4 Institute of Hospital Management, Central South University, Changsha, 410078 China; 5 Department of Pharmacy, Second People’s Hospital of Huaihua City (The Central Hospital of Huaihua City), Jingzhou District, Huaihua, 418400 China; 6 Department of Pharmacy, The Second Affiliated Hospital of Guangzhou Medical University, Guangzhou, 510260 China; 7 Department of Pharmacy, Renmin Hospital, Wuhan University, Wuhan, 430060 China; 8 Department of Pharmacy, the First Affiliated Hospital of Nanchang University, Nanchang, 330006 China; 9 Department of Pharmacy, The People’s Hospital of Liuyang, Liuyang, 410300 China; 10 Changsha Medical University, Changsha, 410219 China

**Keywords:** Carbapenem-resistant *Klebsiella pneumoniae* (CRKP), Polymyxin B (PMB), Ceftazidime/avibactam (CAZ/AVI), Clinical efficacy, Microbiological clearance, Mortality

## Abstract

**Objectives:**

In this retrospective observational multicenter study, we aimed to assess efficacy and mortality between ceftazidime/avibactam (CAZ/AVI) or polymyxin B (PMB)-based regimens for the treatment of Carbapenem-resistant *Klebsiella pneumoniae* (CRKP) infections, as well as identify potential risk factors.

**Methods:**

A total of 276 CRKP-infected patients were enrolled in our study. Binary logistic and Cox regression analysis with a propensity score-matched (PSM) model were performed to identify risk factors for efficacy and mortality.

**Results:**

The patient cohort was divided into PMB-based regimen group (*n* = 98, 35.5%) and CAZ/AVI-based regimen group (*n* = 178, 64.5%). Compared to the PMB group, the CAZ/AVI group exhibited significantly higher rates of clinical efficacy (71.3% vs. 56.1%; *p* = 0.011), microbiological clearance (74.7% vs. 41.4%; *p* < 0.001), and a lower incidence of acute kidney injury (AKI) (13.5% vs. 33.7%; *p* < 0.001). Binary logistic regression revealed that the treatment duration independently influenced both clinical efficacy and microbiological clearance. Vasoactive drugs, sepsis/septic shock, APACHE II score, and treatment duration were identified as risk factors associated with 30-day all-cause mortality. The CAZ/AVI-based regimen was an independent factor for good clinical efficacy, microbiological clearance, and lower AKI incidence.

**Conclusions:**

For patients with CRKP infection, the CAZ/AVI-based regimen was superior to the PMB-based regimen.

**Supplementary Information:**

The online version contains supplementary material available at 10.1007/s15010-024-02324-8.

## Introduction

Bacterial drug resistance has become a global public health threat, among which carbapenem-resistant Enterobacterales (CRE) presents sporadic, outbreaks and epidemics in most countries of the world. CRE was rated by the World Health Organization as the “critical” group of bacterial infections that pose the greatest threat to human health [1]. The threat posed by CRE has proven to be formidable, and the high mortality rate of CRE worldwide is one of the reasons why the epidemiological monitoring of CRE is of great concern [2]. In South Africa, the in-hospital mortality rate associated with CRE bacteremia from 2015 to 2018 was as high as 38% [3]. Among CRE, carbapenem-resistant *Klebsiella pneumoniae* (CRKP) is the most common bacterial species. The hypervirulent of CRKP has made it an internationally concerning pathogen with significant mortality [4]. In a study of 991 patients with CRKP collected from 71 hospitals worldwide during the period from 2017 to 2018, the 30-day mortality in patients with CRKP bacteremia was 34% [5]. Multiple sites can be infected by Klebsiella pneumoniae, including the lung, urinary tract, bloodstream, wounds or surgical sites, and the brain [4].

Therapy options for CRKP are limited, with older treatments such as aminoglycosides, polymyxins, glycylcycline, and fosfomycin showing effectiveness occasionally in vitro. However, these treatments come with adverse effects such as nephrotoxicity and neurotoxicity for polymyxins like colistin and polymyxin B (PMB) [6]. Ceftazidime-avibactam (CAZ/AVI) is composed of the cephalosporin ceftazidime and the novel non-β-lactam β-lactamase inhibitor (BLI) avibactam. Studies have shown that CAZ/AVI has excellent activity against many important Gram-negative pathogens in vitro, such as Ambler class A- (including *Klebsiella pneumoniae* carbapenemase (KPC)), class C-, and some class D β-lactamase enzyme-producers, but has no activity against Metallo-β-lactamase (MBL)-producing strains [7, 8]. CAZ/AVI is approved by the U.S. Food and Drug Administration, China Food and Drug Administration, and the European Medicines Agency for treating complicated intra-abdominal infections, complicated urinary infections, and hospital-acquired pneumonia [9].

In China, the available drugs that cover CRKP are extremely limited, including polymyxins (colistimethate, polymyxin B, and colistin sulfate), tigecycline, and CAZ/AVI. There is disagreement as to which drug treatment regimen is best for CRKP. Most studies have compared the clinical outcomes of CAZ/AVI with colistimethate-based therapy, which is an inactive prodrug of colistin. To our knowledge, few multicenter studies have compared the effectiveness of CAZ/AVI and PMB for CRKP infection [10–12]. In addition, limited data exists comparing CAZ/AVI and PMB, or their combinations, in CRKP infection among Chinese populations. This multicenter real-world study aims to evaluate the efficacy of CAZ/AVI and PMB-based regimens in patients infected with CRKP and identify risk factors for clinical efficacy, microbiological efficacy, 30-day all-cause mortality, and acute kidney injury (AKI).

## Patients and methods

### Ethics

The study was conducted according to the ethical standards of the Helsinki Declaration (1964). Approval for the study protocol was obtained from the Ethics Committees of the Second Xiangya Hospital of Central South University (LYF-2020021) as well as other ethics committees at each participating study site. Due to the retrospective and observational nature of the study, the need for written informed consent was waived.

### Patients

This multicenter retrospective study included patients admitted to the following hospitals between September 2019, and December 2022: Second Xiangya Hospital of Central South University, Xiangya Hospital of Central South University, the First Affiliated Hospital of Nanchang University, the Second Affiliated Hospital of Guangzhou Medical University, and Renmin Hospital of Wuhan University. The inclusion criteria were as follows: (1) CRKP-infected patients confirmed by bacterial culture and drug sensitivity; (2) patients who received CAZ/AVI or PMB-based regimens for treatment ≥ 72 h; and (3) patients with infection-related indicators available to evaluate treatment effectiveness. The exclusion criteria were as follows: (1) patients under the age of 16; (2) Patients in whom effectiveness of PMB or CAZ/AVI treatment at the end of therapy could not be assessed due to the lack of regular evaluation of infection symptoms, reexamination of infection indicators, and identification of pathogens; (3) patients who are simultaneously infected with other non-Enterobacterales gram-negative bacteria (*Acinetobacter baumannii*, *Pseudomonas aeruginosa*, and *Stenotrophomonas maltophilia*); (4) Combination of CAZ/AVI and PMB in the treatment of patients with CRKP infection; (5) Patients whose dose of PMB was less than 1.25 mg/kg intravenously every 12 h.

### Clinical data collection

Data were retrospectively extracted from electronic records on demographics, clinical characteristics, and microbiology, including age, sex, baseline comorbidities, Acute Physiology and Chronic Health Evaluation II (APACHE II) score, infection sites, details of antibiotic use, inflammatory indicators, etc. All the data collected were anonymized.

### Outcomes and definitions

The primary outcomes include 30-day all-cause mortality, microbiological clearance, and clinical success rate. Thiry-day all-cause mortality was defined as the occurrence of death from any cause or patients dying within 30 days. A microbiological clearance was determined by the absence of the initially isolated pathogen from every site of infection in at least two bacterial cultures. For patients with multi-site CRKP infection, bacterial clearance was defined as the absence of further detection of CRKP at all infection sites. Patients who did not have repeated bacterial cultures were excluded from the microbial efficacy evaluation cohort. Clinical success was defined as the achievement of the following criteria in patients with CRKP infection at the end of CAZ/AVI or PMB treatment: absence of the need for vasoactive drugs (such as dopamine, norepinephrine, epinephrine), maintenance of hemodynamic stability, body temperature below 37.3℃, and a significant decrease in infection indicators (including white blood cell count < 12 × 10^9^/L, C-reactive protein, and procalcitonin levels). The assessment of clinical success is conducted by clinicians and pharmacists. Acute kidney injury (AKI) was defined according to the Kidney Disease: Improving Global Outcomes (KDIGO) criteria, which require either a 0.3 mg/dL increase in serum creatinine within 48 h or a 50% increase in serum creatinine within seven days. PMB-based regimens referred to patients who received PMB along with other regimens except for CAZ/AVI, whereas CAZ/AVI-based regimens referred to patients who received CAZ/AVI along with other regimens except for PMB. Preemptive therapy refers to the administration of PMB or CAZ/AVI treatment based on the hospital’s epidemiological situation, after the identification of the pathogen but before the drug sensitivity results are available. Multi-site infection is defined as the presence of symptoms indicating infection in multiple sites during treatment, along with the detection of CRE pathogens in these sites. Pulmonary CRKP infection is defined as the presence of new pulmonary imaging changes, increased sputum production, or other infection-related abnormalities in patients with confirmed detection of pulmonary CRKP.

### Microbiology

*Klebsiella pneumoniae* was identified using a matrix-assisted laser desorption/ ionization-time of flight mass spectrometer (bioMérieux, Marcyl’Étoile, France). Antimicrobial susceptibility testing, including determination of minimum inhibitory concentration (MIC) breakpoints for meropenem, imipenem, colistin, tigecycline, and ceftazidime was performed using the broth microdilution method with the VITEK®2 system (bioMérieux, Marcy-l’Étoile, France). Interpretation of antimicrobial susceptibilities followed the guidelines established by the Clinical and Laboratory Standard Institute (CLSI) in 2020 [13]. Specifically, the MIC breakpoints for tigecycline and colistin/PMB were in accordance with the criteria provided by the European Committee on Antimicrobial Susceptibility Testing (EUCAST) in 2020 [14]. The modified Hodge test with ethylene diamine tetraacetic acid disk test was used to detect carbapenemase-producing Enterobacterales phenotypically [15, 16]. According to the results of antimicrobial susceptibility testing, the patients were treated with the susceptible drugs.

### Statistical analysis

Statistical analysis was conducted using SPSS 25.0 (IBM, Armonk, NY, USA). Quantitative data with normal or non-normal distribution were represented as mean ± standard deviation and median (interquartile range [IQR]). Normally distributed continuous variables were evaluated by ANOVA and t-test, while non-normally distributed continuous variables were assessed using nonparametric test (Mann-Whitney U test). Categorical data were expressed as frequencies and percentages, and group comparisons were performed using the Chi-square test or Fisher’s exact test. Propensity score-matched (PSM), at a ratio of 1:1, was employed using the PMB group and CAZ/AVI group as indicator variables. Variables with *p* < 0.1 in the univariate analysis of the PMB group and CAZ/AVI groups were selected as predictors for matching. A match tolerance value of 0.2 was chosen, and case order was randomized during matching to obtain matched data. Univariate analysis was used to analyze the data before PSM. Factors with *p* < 0.1 from the data before PSM were included in multivariate logistic regression analysis and Cox regression analysis. Multivariate logistic regression and Cox logistic regression analysis were used to assess potential independent factors of efficacy and mortality. Survival analysis was performed using the Kaplan-Meier method and Cox logistic regression analysis, with pairwise comparisons determined by the log-rank test. *P* < 0.05 was considered statistically significant.

## Results

### Baseline clinical characteristics of patients

**Fig. 1:**
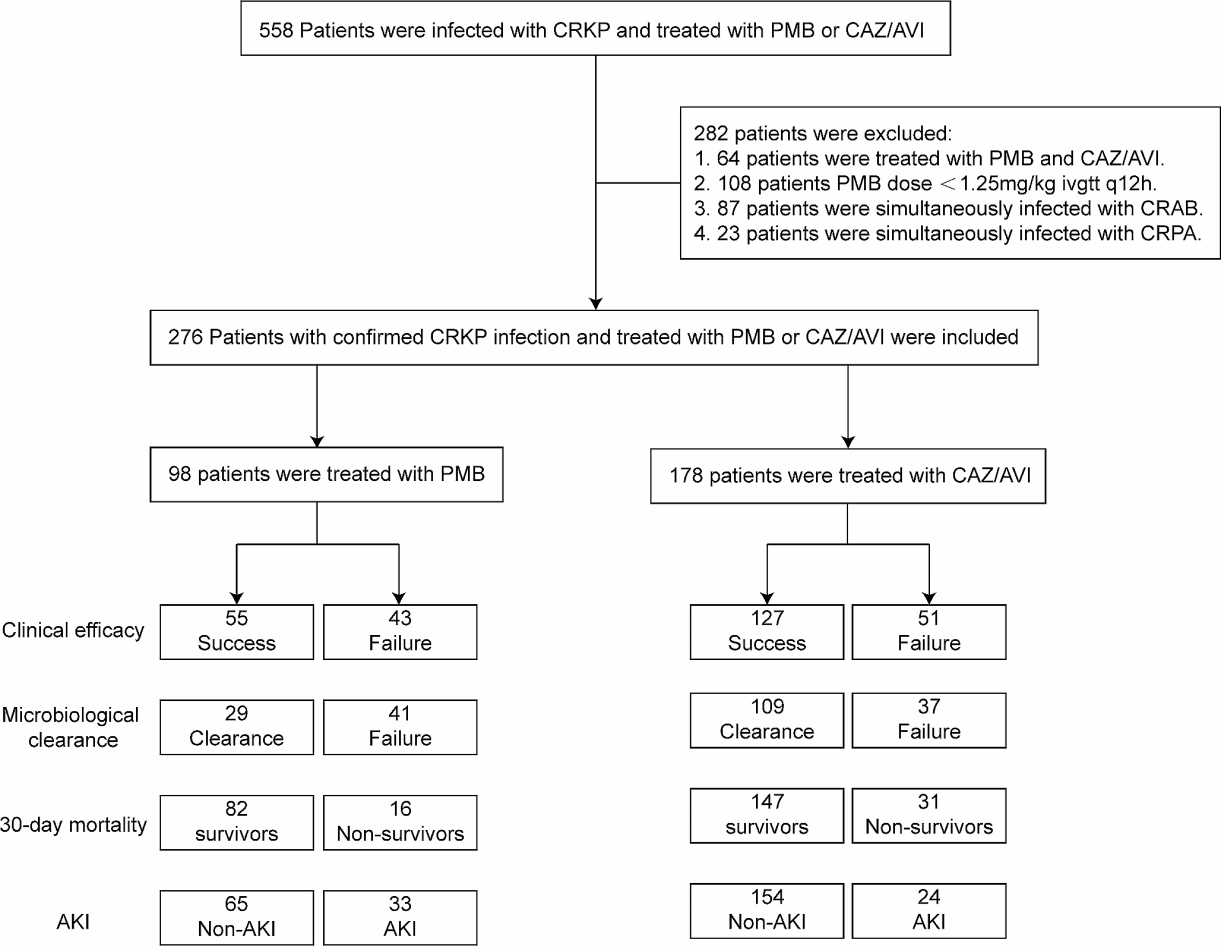
Flow diagram depicting the of inclusion and exclusion criteria for patients

After screening patients from five hospitals based on inclusion and exclusion criteria, a total of 276 CRKP-infected patients were enrolled in this multicenter real-world study (Fig. 1). The patients were divided into PMB-based group (*n* = 98, 35.5%) and CAZ/AVI-based group (*n* = 178, 64.5%).

The baseline clinical characteristics and demographics of patients are summarized in Table 1. The median age of patients was 58.5 (46.0–68.0) years, with males accounting for 69.6% of the total. Multi-site infections were observed in 33% of the cases. The most common infection site was the respiratory tract (76.4%), followed by bloodstream infections (27.9%), urinary system infections (17.8%), abdominal infections (14.9%), skin and soft tissue infections (4.7%), and central nervous system infections (1.4%). The baseline levels of creatinine and creatinine clearance rate (CCR) before treatment were 91.2 (57.0-166.2) µmol/L and 60.6 (32.8-100.4) mL/min, respectively. Continuous renal replacement therapy (CRRT) and intermittent renal replacement therapy (IRRT) were used in 9.8% of the cases (Table 1).

**Table 1:** Demographics and clinical characteristics of CRKP-infected patients treated with different regimens

Demographics and clinical characteristics	Before PSM					After PSM				
	Total (*N* = 276)	PMB (*N* = 98)	CAZ/AVI (*N* = 178)	SMD	*P*-value	Total (*N* = 126)	PMB (*N* = 63)	CAZ/AVI (*N* = 63)	SMD	*P*-value
**Demographic characteristics**										
Age(years)	58.5(46.0–68.0)	56.5(45.0–68.0)	59.0(47.0-69.3)	0.236	0.135	56.0(46.0–68.0)	57.0(46.0–69.0)	52.0(45.0–68.0)	0.167	0.289
Gender (male)	192(69.6%)	61(62.2%)	131(73.6%)	0.260	**0.050**	80(63.5%)	40(63.5%)	40(63.5%)	<0.001	>0.999
Baseline creatinine (µmol/L)	91.2(57.0-166.2)	82.9(52.6-139.1)	104.0(60.7-183.2)	0.102	**0.047**	77.0(48.6–139.0)	72.0(48.0-116.4)	82.0(50.1–172.0)	0.142	0.439
Baseline CCR (mL/min)	60.6(32.8-100.4)	69.5(37.3-117.3)	52.3(27.4–92.1)	0.231	**0.038**	79.8(38.7-127.6)	82.4(47.1-133.6)	79.5(29.3-115.5)	0.094	0.373
RRT	27(9.8%)	13(13.3%)	14(7.9%)	0.168	0.148	13(10.3%)	8(12.7%)	5(7.9%)	0.164	0.380
Mechanical ventilation	163(59.1%)	59(60.25)	104(58.4%)	0.041	0.774	75(59.5%)	40(63.5%)	35(55.6%)	0.142	0.364
Vasoactive drugs	132(47.8%)	50(51.0%)	82(46.1%)	0.100	0.431	54(42.9%)	30(47.6%)	24(38.1%)	0.201	0.280
ICU administration	192(69.6%)	66(67.3%)	126(70.8%)	0.087	0.552	84(66.7%)	43(68.3%)	41(65.1%)	0.063	0.705
Sepsis/Septic shock	111(40.2%)	45(45.9%)	66(37.1%)	0.183	0.152	52(41.3%)	26(41.3%)	26(41.3%)	0.000	>0.999
Hospital stay (days)	31.0(20.0-50.8)	28.0(16.0–48.0)	33.5(21.0–52.0)	0.054	0.127	30.5(20.0-47.5)	25.0(16.0–46.0)	35.0(23.0–52.0)	0.152	0.065
APACHE II score	24.1 ± 8.5	22.1 ± 5.4	25.3 ± 9.7	0.379	**0.002**	23.0 ± 7.2	22.5 ± 4.9	23.5 ± 9.0	0.136	0.448
**Comorbidity**										
Solid organ transplantation	24(8.7%)	7(7.1%)	17(9.6%)	0.106	0.497	11(8.7%)	5(7.9%)	6(9.5%)	0.071	0.752
Hypoproteinemia	105(38.0%)	32(32.7%)	73(41.0%)	0.165	0.171	49(38.9%)	22(34.9%)	27(42.9%)	0.164	0.361
Renal insufficiency	53(19.2%)	14(14.3%)	39(21.9%)	0.203	0.124	22(17.5%)	9(14.3%)	13(20.6%)	0.184	0.348
Diabetes mellitus	78(28.3%)	17(17.3%)	61(34.3%)	0.375	**0.003**	30(23.8%)	14(22.2%)	16(25.4%)	0.070	0.676
Digestive system diseases	133(48.2%)	48(49.0%)	85(47.8%)	0.025	0.845	64(50.8%)	29(46.0%)	35(55.6%)	0.199	0.285
Cerebrovascular diseases	103(37.3%)	35(35.7%)	68(38.2%)	0.051	0.683	44(34.9%)	25(39.7%)	19(30.2%)	0.209	0.262
Cardiovascular diseases	161(58.3%)	48(49.0%)	113(63.5%)	0.294	**0.019**	65(51.6%)	33(52.4%)	32(50.8%)	0.020	0.859
Malignancy	45(16.3%)	11(11.2%)	34(19.1%)	0.213	0.090	22(17.5%)	7(11.1%)	15(23.8%)	0.341	0.060
***Infection sites***										
Multi-site infection	91(33.0%)	19(19.4%)	72(40.4%)	0.447	**<0.001**	44(34.9%)	19(30.2%)	25(39.7%)	0.209	0.262
Respiratory tract	211(76.4%)	67(68.4%)	144(80.9%)	0.019	**0.019**	93(73.8%)	48(76.2%)	45(71.4%)	0.113	0.543
Blood	77(27.9%)	26(26.5%)	51(28.7%)	0.066	0.707	39(31.0%)	16(25.4%)	23(36.5%)	0.259	0.177
Abdominal	41(14.9%)	15(15.3%)	26(14.6%)	0.106	0.876	23(18.3%)	12(19.0%)	11(17.5%)	0.052	0.818
Urinary tract	49(17.8%)	11(11.2%)	38(21.3%)	0.068	**0.035**	22(17.5%)	9(14.3%)	13(20.6%)	0.184	0.348
Central nervous system	4(1.4%)	1(1.0%)	3(1.7%)	0.170	1.000	2(1.6%)	0(0.0%)	2(3.2%)	0.240	0.496
Skin and soft tissue	13(4.7%)	2(2.0%)	11(6.2%)	0.117	0.209	7(5.6%)	2(3.2%)	5(7.9%)	0.217	0.437
***Pathogenic bacteria***										
Only CRKP infection	268(97.1%)	95(96.9%)	173(97.2%)	0.015	1.000	121(96.0%)	60(95.2%)	61(96.8%)	0.102	0.648
CRKP + Other CREs	8(2.9%)	3(3.1%)	5(2.8%)	0.015	1.000	5(4.0%)	3(4.8%)	2(3.2%)	0.102	>0.999
**Antibiotic regimens**										
Treatment duration(days)	10.0(7.0–14.0)	8.8(6.0–14.0)	10.0(7.0–14.0)	0.179	0.189	8.5(6.0-13.3)	8.5(6.0–13.0)	8.0(6.0–14.0)	0.093	0.897
Combined antibiotics	1.0(0.0–1.0)	1.0(1.0–2.0)	1.0(0.0–1.0)	0.712	**<0.001**	1.0(0.0–1.0)	1.0(1.0–2.0)	1.0(0.0–1.0)	0.254	0.108
Monotherapy	100(36.2%)	12(12.2%)	88(49.4%)	0.772	**<0.001**	34(27.0%)	12(19.0%)	22(34.9%)	0.359	0.070
Preemptive therapy	78(28.3%)	17(17.3%)	61(34.3%)	0.375	**0.003**	32(25.4%)	11(17.5%)	21(33.3%)	0.366	0.065
+ SMZ	10(3.6%)	1(1.0%)	9(5.1%)	0.216	0.167	1(0.8%)	1(1.6%)	0(0.0%)	0.225	>0.999
+ Quinolones	21(7.6%)	7(7.1%)	14(7.9%)	0.027	0.829	11(8.7%)	6(9.5%)	5(7.9%)	0.071	0.752
+ Aminoglycosides	16(5.8%)	7(7.1%)	9(5.1%)	0.089	0.478	7(5.6%)	5(7.9%)	2(3.2%)	0.217	0.437
+ *β-lactam*	39(14.1%)	25(25.5%)	14(7.9%)	0.506	**<0.001**	23(18.3%)	14(22.2%)	9(14.3%)	0.206	0.249
+ Tigecycline	53(19.2%)	28(28.6%)	25(14.0%)	0.368	**0.003**	29(23.0%)	16(25.4%)	13(20.6%)	0.095	0.525
+ Carbapenem	87(31.5%)	56(57.1%)	31(17.4%)	0.854	**<0.001**	49(38.9%)	27(42.9%)	22(34.9%)	0.164	0.361
**Efficacy and mortality**										
Clinical efficacy	182(65.9%)	55(56.1%)	127(71.3%)	0.315	**0.011**	81(64.3%)	32(50.8%)	49(77.8%)	0.561	**0.002**
7-day microbiological clearance	75/214(35.0%)	18/78(23.1%)	57/136(41.9%)	0.397	**0.005**	35/101(34.7%)	10/48(20.8%)	25/53(47.2%)	0.544	**0.005**
Microbiological clearance	138/216(63.9%)	29/70(41.4%)	109/146(74.7%)	0.706	**<0.001**	60/102(58.8%)	17/45(37.8%)	43/57(75.4%)	0.747	**<0.001**
30-day mortality	47(17.0%)	16(16.3%)	31(17.4%)	0.026	0.818	25(19.8%)	13(20.6%)	12(19.0%)	0.050	0.823
AKI	57(20.7%)	33(33.7%)	24(13.5%)	0.517	**<0.001**	32(25.4%)	24(38.1%)	8(12.7%)	0.572	**0.001**
Bacterial removal time (days)	8.0(6.0–12.0)	9.0(6.0-11.8)	5.0(6.0–12.0)	0.022	0.725	7.5(5.3–11.0)	7.5(5.3–11.0)	8.0(6.0–12.0)	0.040	0.837
Survival time (days)	30.0(24.3–30.0)	30.0(30.0–30.0)	30.0(18.0-30.8)	0.027	0.016	30.0(20.5–30.0)	30.0(30.0–30.0)	30.0(13.0–30.0)	0.217	0.229

Vasoactive drugs include norepinephrine, dopamine, epinephrine, isoproterenol, phentolamine, and nitroglycerin; ICU, Intensive Care Unit; APACHE II, Acute Physiology and Chronic Health Evaluation II; CR-GNB, Carbapenem-resistant Gram-negative Bacteria; CAZ/AVI, ceftazidime/avibactam, AKI, acute kidney injury; CCR, creatinine clearance rate; SMZ, Sulfamethoxazole and trimethoprim; RRT, renal replacement therapy; PSM, propensity score-matched; SMD, standardized mean difference. Bold font indicates data with significant differences

There was no difference between the two regimen groups on demographic characteristics such as age, mechanical ventilation, and use of vasoactive drugs. However, baseline creatinine and CCR were higher in the CAZ/AVI group than in the PMB group (*p* < 0.05). The incidence of multi-site infection was higher in the CAZ/AVI group compared to the PMB group (40.4% vs. 19.4%; *p* < 0.001). Preemptive therapy was more frequently used in the CAZ/AVI group than in the PMB group (34.3% vs. 17.3%; *p* = 0.019). The details regarding the usage of PMB and CAZ/AVI can be found in Table S1. The APACHE II score was higher in the CAZ/AVI group compared to the PMB group [(25.3 ± 9.7) vs. (22.1 ± 5.4); *p* = 0.002].

Sixty-three pairs of patients treated with PMB or CAZ/AVI were matched according to propensity scores. After PSM model, notable differences were observed in clinical efficacy (50.8% vs. 77.8%; *p* = 0.002), 7-day microbiological clearance (20.8% vs. 47.2%; *p* = 0.005), microbiological clearance (37.8% vs. 75.4%; *p* < 0.001), and AKI (38.1% vs. 12.7%; *p* = 0.001) between the PMB group and the CAZ/AVI group. However, there was no significant difference in 30-day mortality between the two treatment groups (20.6% vs. 19.0%; *p* = 0.823) (Table 1).

Due to the high prevalence of patients with pulmonary infection and bloodstream infections, we conducted a subgroup analysis. Regarding CRKP pulmonary infection, the real-world data revealed that the clinical treatment success rate of the PMB group was slightly lower than that of the CAZ/AVI group, but the difference was not statistically significant (58.2% vs. 67.4%; *p* = 0.196). However, after PSM, a significant difference was observed (52.1% vs. 75.6%; *p* = 0.019). Regarding CRKP pulmonary infection, both the real-world data and PSM data indicated that the microbiological clearance rate in the PMB group was lower than that in the CAZ/AVI group (40.0% vs. 76.3%; *p* < 0.001). In terms of the incidence of AKI, both the real-world data and PSM data demonstrated a higher occurrence in the PMB group compared to the CAZ/AVI group (38.8% vs. 13.2%; *p* < 0.001). However, surprisingly, there was no significant difference in 30-day mortality between the two groups (13.4% vs. 18.1%, *p* = 0.401) (Table S2). In the subgroup analysis of bloodstream infection, the PMB group exhibited lower clinical treatment success rate and microbial clearance rate compared to the CAZ/AVI group (46.2%vs. 76.5%, 33.3% vs. 77.8%; both *p* < 0.05). Nonetheless, no significant difference was observed in terms of mortality and the incidence of AKI (Table S3).

### Microbiological culture and antibiotic susceptibility characteristics

Bacterial culture and drug sensitivity tests confirmed that 276 patients were infected with CRKP. Among these patients, 268 (97.1%) patients were only infected by CRKP and 8 (2.9%) patients were infected by CRKP and other CRE strains. Drug sensitivity results showed that all strains were carbapenem-resistant. Among the 284 CRE strains, 97.2% were identified as *Klebsiella Pneumoniae*, while the remaining strains were classified as *Escherichia coli* and *Enterobacter cloacae*. A significant proportion of *Klebsiella Pneumoniae* isolates exhibited resistance to amikacin (81.2%) and sulfamethoxazole (78.6%), but showed susceptibility to CAZ (90.2%), polymyxins (85.5%), and tigecycline (59.8%) (Table S4). Out of the cases included, we detected drug resistance mechanisms in only 44 strains of CRKP. Among them, 41 strains secreted KPC, and 3 strains secreted metallo-β-lactamases.

### Comparison of efficacy and mortality between PMB and CAZ/AVI on CRKP-infected patients and identification of risk factors

**Fig. 2:**
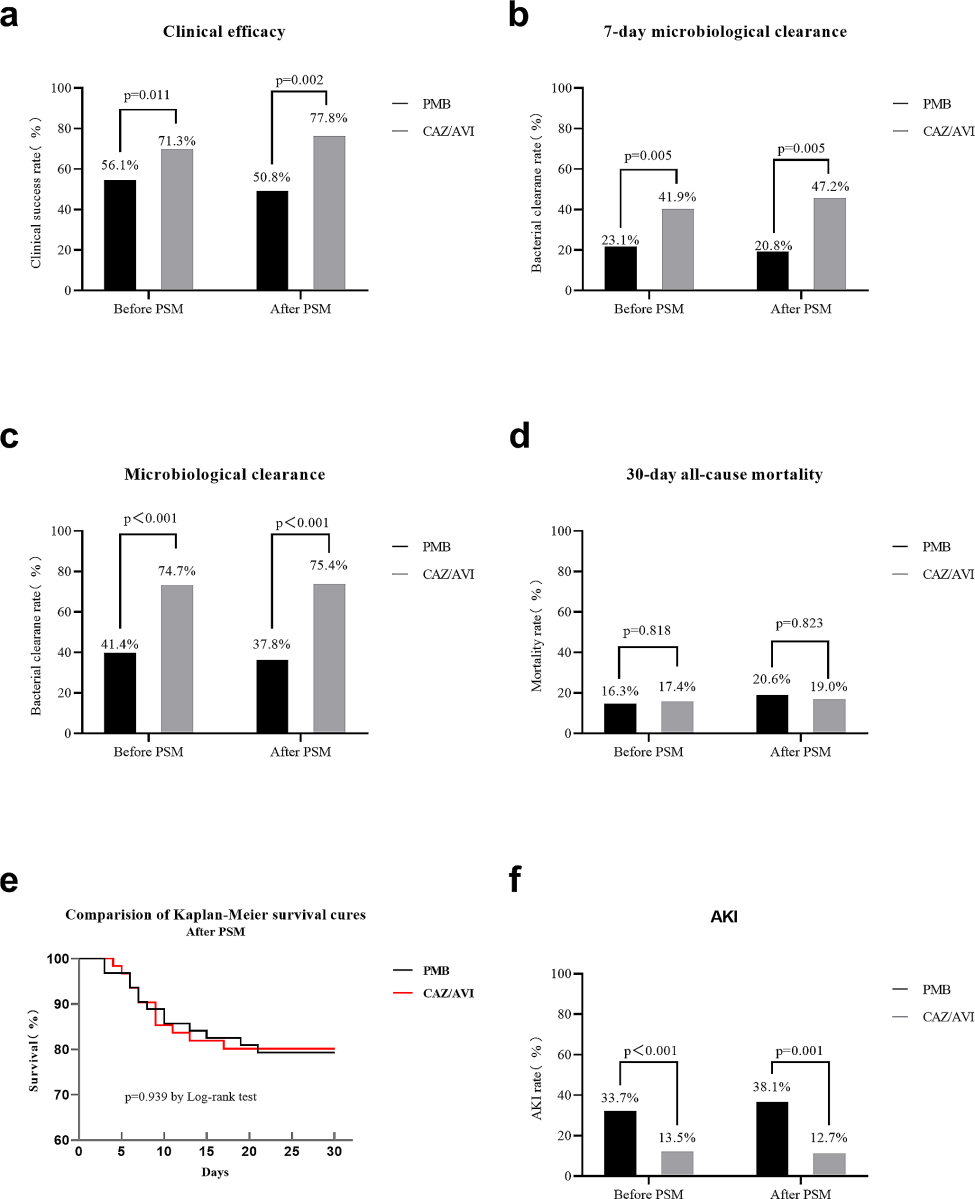
Efficacy and mortality comparison between PMB and CAZ/AVI for CRKP-infected patients. (**a**) Clinical efficacy comparison; (**b**) 7-day microbiological clearance comparison; (**c**) Microbiological clearance comparison; (**d**) 30-day all-cause mortality comparison; (**e**) Kaplan-Meier survival curves for PSM patients; (**f**) AKI comparison

The rate of clinical efficacy was significantly higher in the CAZ/AVI group than in the PMB group (71.3% vs. 56.1%; *p* = 0.011) (Fig. 2a). To identify factors associated with clinical efficacy, we performed univariate and multivariable analysis. Patients were divided into clinical failure and success groups, and their demographic and clinical characteristics were compared (Table S5). After calculating propensity scores and incorporating them into regression analysis, we found that the treatment duration [OR = 0.848 (0.769–0.936), *p* = 0.001] was an independent factor associated with clinical efficacy. Additionally, CAZ/AVI-based regimens [OR = 0.237 (0.100-0.563), *p* = 0.001] were found to be independently favorable for clinical efficacy compared to PMB-based regimens (Table 2).

**Table 2:** Logistic regressive analysis of factors associated with clinical efficacy, microbiological clearance, and AKI

Demographics and clinical characteristics	Before PSM			After PSM		
	B value	OR(95%CI)	*P* -value	B value	OR(95%CI)	*P* -value
**Clinical efficacy**						
Mechanical ventilation	0.939	2.556(1.400-4.668)	**0.002**	0.441	1.554(0.621–3.886)	0.346
Sepsis/Septic shock	0.576	1.778(1.008–3.137)	**0.047**	0.269	1.308(0.540–3.173)	0.552
APACHE II score	0.067	1.069(1.032–1.107)	**<0.001**	0.049	1.050(0.989–1.115)	0.113
Treatment duration (days)	-0.052	0.949(0.902–0.998)	**0.042**	-0.165	0.848(0.769–0.936)	**0.001**
CAZ/AVI-based regimens: Compared with PMB-based regimens	-0.889	0.411(0.230–0.735)	**0.003**	-1.438	0.237(0.100-0.563)	**0.001**
**Microbiological clearance**						
Solid organ transplantation	-2.517	0.081(0.010–0.678)	**0.020**	-1.876	0.153(0.016–1.474)	0.104
Treatment duration (days)	-0.096	0.909(0.854–0.968)	**0.003**	-0.136	0.873(0.790–0.964)	**0.007**
CAZ/AVI-based regimens: Compared with PMB-based regimens	-1.613	0.199(0.103–0.385)	**<0.001**	-2.000	0.135(0.051–0.359)	**<0.001**
**AKI**						
+β-lactam	0.288	1.334(0.603–2.951)	0.477	0.692	1.998(0732-5.452)	0.177
CAZ/AVI-based regimens: Compared with PMB-based regimens	-1.130	0.323(0.174–0.599)	**<0.001**	-1.403	0.246(0.099–0.608)	**0.002**

The 7-day microbiological clearance rates were 23.1% in the PMB group and 41.9% in the CAZ/AVI group, showing a significant difference (*p* = 0.005) (Fig. 2b). After treatment, the microbiological clearance rate reached 41.4% in the PMB group and 74.7% in the CAZ/AVI group (*p* < 0.001) (Fig. 2c). The 216 CRKP-infected patients who had their microbiological clearance evaluated after treatment were divided into failure and clearance groups, and their demographic and clinical characteristics were compared (Table S6). Binary logistic regression analysis using a PSM model indicated that the treatment duration [OR = 0.873 (0.790–0.964), *p* = 0.007] was an independent factor associated with microbiological clearance. Moreover, CAZ/AVI-based regimens [OR = 0.135 (0.051–0.359), *p* < 0.001] were independent favorable factors to microbiological clearance compared to PMB-based regimens (Table 2).

The 30-day all-cause mortality rate did not differ significantly between patients treated with PMB (16.3%) and CAZ/AVI (17.4%, *p* = 0.818) (Fig. 2d). According to the result of Kaplan-Meier survival analysis, there was no significant difference in 30-day mortality between the two treatment groups (Fig. 2e). We compared the demographics and clinical characteristics of 229 survivors and 47 non-survivors. Univariate analysis results revealed differences in age (*p* < 0.001), baseline CCR (*p* = 0.045), mechanical ventilation (*p* = 0.002), vasoactive drugs use (*p* < 0.001), sepsis/septic shock (*p* < 0.001), hospital stay (days) (*p* = 0.023), APACHE II score (*p* < 0.001), and treatment duration (*p* = 0.001) between the two groups (Table S7). Cox regression analysis, using a PSM model, found that vasoactive drugs [HR = 2.431 (1.011–5.487), *p* = 0.047], sepsis/septic shock [HR = 3.726 (1.505–9.221), *p* = 0.004], APACHE II score [HR = 1.062 (1.012–1.114), *p* = 0.014], and treatment duration [HR = 0.819 (0.726–0.923), *p* = 0.001] were risk factors for 30-day all-cause mortality (Table 3).

**Table 3:** Cox regressive analysis of factors associated with 30-day all-cause mortality

Demographics and clinical characteristics	Before PSM			After PSM		
	B value	HR(95%CI)	*P* -value	B value	HR(95%CI)	*P* -value
Age(years)	0.025	1.026(1.006–1.046)	**0.010**	0.008	1.008(0.980–1.038)	0.563
Vasoactive drugs	0.886	2.426(1.204–4.889)	**0.013**	0.888	2.431(1.011–5.847)	**0.047**
Sepsis/Septic shock	0.811	2.251(1.213–4.177)	**0.010**	1.315	3.726(1.505–9.221)	**0.004**
APACHE II score	0.047	1.048(1.021–1.076)	**0.001**	0.060	1.062(1.012–1.114)	**0.014**
Treatment duration (days)	-0.113	0.893(0.834–0.957)	**0.001**	-0.200	0.819(0.726–0.923)	**0.001**

Among 276 CRKP-infected patients who received PMB-based or CAZ/AVI-based regimens, 57 (20.7%) developed AKI, with a significantly higher incidence in the PMB group compared to the CAZ/AVI group (33.7% vs. 13.5%, *p* < 0.001) (Fig. 2f). We assessed the potential risk factors for AKI in CRKP-infected patients. The results of the univariate analysis showed that PMB-based regimens were associated with AKI (*p* < 0.001) (Table S8). Logistic regressive analysis, using a PSM model, confirmed that PMB-based regimens [OR = 0.246 (0.099–0.608), *p* = 0.002] were associated with a higher risk of AKI (Table 2).

## Discussion

In recent years, the incidence rate of CRE has sharply increased, becoming a global public health problem. Among the different kinds of CRE, CRKP is associated with significant mortality [17]. Data from the China Antimicrobial Surveillance Network (CHINET) have shown a notable increase in the prevalence of CRKP in China since 2005, rising from 3.0 to 24.4% in 2021. In China, limited treatment options are available for CRKP infections, with tigecycline, polymyxins, and CAZ/AVI becoming commonly used [6]. However, it remains unclear which option is superior for treating patients infected with CRKP, necessitating further research. In the United States and Europe, CAZ/AVI has been approved for the treatment of complex urinary tract infections (cUTI), including pyelonephritis, complex intra-abdominal infections (cIAI), and hospital-acquired pneumonia (HAP), including ventilator-associated pneumonia (VAP) [9]. To date, CAZ/AVI has been approved in over 40 countries and regions around the world, and China approved its use in May 2019. CAZ-AVI is well tolerated by healthy individuals and hospitalized patients, with mostly mild or moderate side effects [18, 19]. Numerous studies have examined the effectiveness of CAZ/AVI and polymyxins and have consistently demonstrated the considerable value of CAZ/AVI in treating CRKP infections. Studies have shown that patients treated with CAZ-AVI or colistin for CRE infections have a 30-day mortality rate of 9% and 32%, respectively [20]. A previous study compared the efficacy of CAZ/AVI and PMB in 105 patients with CRKP infection. The findings revealed that patients receiving CAZ/AVI had significantly lower 28-day mortality rates, higher rates of microbiological eradication, and 28-day clinical success compared to those treated with PMB [11]. The results from our study also showed that patients treated with CAZ/AVI exhibited higher rates of clinical efficacy and microbiological clearance, which was comparable to their findings. In a larger retrospective multicenter study involving 230 patients with CRE infections, Almangour et al. reported that CAZ/AVI was independently associated with clinical cure and lower incidence of AKI [21]. A recent meta-analysis encompassing 11 articles with 1,205 patients demonstrated that CRE bloodstream infection patients treated with CAZ/AVI had a significantly higher clinical cure rate and lower nephrotoxicity rate than those receiving colistin-based regimens [22]. In line with previous reports, our study also observed a lower incidence of AKI with CAZ/AVI treatment. Collectively, our retrospective multicenter cohort study supported the superiority of CAZ/AVI-based regimens over PMB-based regimens for CRKP-infected patients. Current clinical studies suggested that CAZ/AVI is a last-line antibiotic, to be used as a targeted therapy for certain CRE infections.

In the 2021 CHINET data, the resistance rates for meropenem and imipenem among 49,150 *Klebsiella* spp. were 21.9% and 20.8%, respectively. The resistance rate for CAZ/AVI was 7.4% (adult for 6.7%, children for 22.5%) for all *Klebsiella* strains. Recently, there have been increasing reports of resistant or reduced-sensitivity strains to CAZ/AVI. A neonatal intensive care unit in China reported that 23.3% of CRKP strains were resistant to CAZ/AVI [23]. In a large-scale multicenter survey, the resistance rate of CAZ/AVI in CRKP strains was 3.7% [24]. Among the resistant isolates, 53.1% were *Klebsiella pneumoniae* producing metallo-β-lactamase (MBL-KP), 40.6% were *Klebsiella pneumoniae* producing KPC (KPC-KP) and 6.3% produced both MBL and KPC [24]. Most of the CAZ/AVI resistance mechanisms in these strains were attributed to blaKPC mutations, leading to amino acid substitutions in *β*-lactamase and changes in gene expression [25]. Another study found that the resistance to CAZ/AVI was induced under drug-selective pressure, caused by blaKPC-2 overexpression and/or substitutions in the Ω-loop of KPC [26]. Therefore, except for the drug sensitivity test of carbapenem and CAZ/AVI, genotypic identification of CRKP is beneficial to explore the resistance mechanism. It is worth noting that in our study, most CRKP strains were sensitive to CAZ (as high as 90.2%) and polymyxins (85.5%), and only a few CRKP strains showed resistance to these antibiotics. Once CRKP strains become resistant to these “last-line” treatments, limited options are available for patients, and patients may have a poor prognosis. Hence, when using novel antibiotics such as CAZ/AVI, clinicians should carefully consider the “right antibiotic, at the right dose, for the right duration, at the right time” to minimize the emergence of drug resistance and optimize their use in treating CRKP-infected patients [27].

In our study, the 30-day all-cause mortality rate of CRKP-infected patients was 17.0%. Survival analysis revealed no significant difference in survival rate between patients receiving CAZ/AVI-based regimens and those receiving PMB-based regimens. Additionally, there was no significant difference in 30-day mortality between the PMB group and CAZ/AVI group. Further analysis identified independent factors associated with 30-day all-cause mortality, including vasoactive drugs, sepsis/sepsis shock, the APACHE II score, and treatment duration. In a multicenter study evaluating the efficacy of CAZ/AVI in the treatment of gram-negative bacteria infections in critically ill patients, up to 95.5% of patients had sepsis or septic shock and required life-supporting treatment [28]. In our study, the rates of vasoactive drug use and septic/septic shock reached 47.8% and 40.2%, respectively, and they were negative factors for mortality. A single-center cohort study of CAZ/AVI for CRKP infection showed that the Charlson comorbidity index (> 3) was associated with decreased 30-day mortality [29]. Therefore, it is necessary to pay attention to the influence of comorbidities on mortality in critically ill patients during treatment. Regarding clinical efficacy and microbiological clearance, we observed that treatment duration and CAZ/AVI-based regimens were independent factors. Similar findings were reported by Fang et al., who discovered that a CAZ/AVI-based regimen, prior antibiotic use within 90 days, and Charlson comorbidity index (≥ 3) were associated with a lower rate of 28-day bacterial clearance [30]. Another study by Zhou et al. supported our results by demonstrating that shorter durations of antimicrobial therapy led to a worse prognosis compared to longer durations [31]. However, it is important to note that the appropriate antimicrobial treatment duration for infection depends on multiple factors, such as infection severity, multidrug-resistant organisms, and immune status [32–34]. Prolonged antibiotic exposure has been linked to the development of antimicrobial resistance [34]. Thus, our conclusion regarding the treatment duration needs further investigation in different types of CRKP infections to strike a balance between clinical efficacy and side effects.

Our study revealed a higher incidence of AKI in patients treated with PMB-based regimens (33.7%) compared to CAZ/AVI-based regimens (13.5%). Nephrotoxicity, particularly associated with polymyxins like PMB and polymyxin E (colistin), is a serious adverse effect that limits their use [32]. The incidence of polymyxin-associated AKI ranges from 10 to 60%, predominantly due to the combined use of nephrotoxic agents and inappropriate dosing regimens [35–37]. The PMB loading dose and combined use of other nephrotoxic drugs were found to be independent risk factors for AKI [38]. A retrospective cohort study revealed that patients who received colistin for Gram-negative infections had a higher rate of mortality and AKI compared to those who received β-lactam/β-lactamase inhibitors [39]. Our results are consistent with previous studies, suggesting an association between PMB use and AKI. Further research is warranted to identify independent factors for nephrotoxicity associated with PMB and CAZ/AVI in real-world settings.

In this study, we compared the clinical efficacy and safety of CAZ/AVI-based and PMB-based therapeutic regimens in CRKP-infected patients. To control the potential bias, we calculated propensity scores and incorporated them into logistic and Cox regression analysis based on the PSM model. Nevertheless, there were limitations in our study. Firstly, our study was a retrospective observational cohort study and the definition of clinical success is partly subjective, which is prone to produce information bias. Therefore, in the stage of design, it is necessary to have stricter and more objective definitions of outcomes and strive to quantify the indicators. Secondly, the sample size was limited after subgroup analysis based on different regimens. More well-designed studies with a larger number of eligible participants should be performed in the future. Thirdly, grouping patients into only two categories based on regimens might limit the accuracy of evaluating patient efficacy. Further subdividing patients into more medication groups could provide a more accurate assessment of efficacy.

## Conclusion

In patients with CRKP infection, the CAZ/AVI-based regimen was superior to the PMB-based regimen in clinical efficacy, microbiological clearance, and lower incidence of AKI. These observations require further confirmation in larger randomized prospective clinical trials.

## Electronic supplementary material

Below is the link to the electronic supplementary material.

Supplementary Material 1

## Data Availability

No datasets were generated or analysed during the current study.
