# Supplementary material for: Ceftazidime/avibactam versus polymyxin B in carbapenem-resistant Klebsiella pneumoniae infections: a propensity score-matched multicenter real-world study
Source: Infection. 2024 Jun 17;53(1):95–106. doi: 10.1007/s15010-024-02324-8 (PMC11825550; doi:10.1007/s15010-024-02324-8)
Supplement: Supplementary file 1 — Supplementary Material 1 [file 15010_2024_2324_MOESM1_ESM.docx]

**Supplementary Tables**

**Table S1. Details of PMB and CAZ/AVI use**

**Table S2. Subgroup analysis of pulmonary infection**

**Table S3. Subgroup analysis of bloodstream infection**

**Table S4. Antibiotic susceptibility characteristic of 284 CRE strains**

**Table S5. Univariate analysis of factors associated with clinical efficacy in CRKP-infected patients**

**Table S6. Univariate analysis of factors associated with microbiological clearance in CRKP-infected patients**

**Table S7. Factors associated with 30-day all-cause mortality in patients with CRKP infection**

**Table S8. Univariate analysis of factors associated with AKI in CRKP-infected patients**

**Table S1. Details of PMB and CAZ/AVI use**

| **PMB**  **(N=98)** | | **CAZ/AVI**  **(N=178)** | |
| --- | --- | --- | --- |
| Combined atomization administration | 17 (17.3%) | 1.25g qd | 2 (1.0%) |
| Loading dose | 42 (42.9%) | 1.25g q12h | 13 (6.6%) |
| 50mg q12h | 39 (39.8%) | 1.25g q8h | 23 (11.6%) |
| 75mg q12h | 27 (27.6%) | 2.5g qd | 1 (0.5%) |
| 80mg q12h | 1 (1.0%) | 2.5g q12h | 8 (4.0%) |
| 87.5mg q12h | 1 (1.0%) | 2.5g q8h | 130 (65.7%) |
| 90mg q12h | 1 (1.0%) | 2.5g q6h | 1 (0.5%) |
| 100mg q12h | 27 (27.6%) | Reduce the dose during treatment | 8 (4.0%) |
| 125mg q12h | 2 (2.0%) | Increase the dose during treatment | 2 (1.0%) |

**Table S2.** **Subgroup analysis of pulmonary infection**

| Treatment outcome | Before PSM | | | After PSM | | |
| --- | --- | --- | --- | --- | --- | --- |
|  | PMB  (N=67) | CAZ/AVI  (N=144) | p-value | PMB  (N=48) | CAZ/AVI  (N=45) | p-value |
| Clinical efficacy | 39(58.2%) | 97(67.4%) | 0.196 | 25(52.1%) | 34(75.6%) | **0.019** |
| 7-day Microbiological clearance* | 11/53(20.8%) | 48/110(43.6%) | **0.004** | 9/38(23.7%) | 20/37(54.1%) | **0.007** |
| Microbiological clearance* | 20/50(40.0%) | 90/118(76.3%) | **＜0.001** | 16/37(43.2%) | 33/41(80.5%) | **0.001** |
| 30-day mortality | 9(13.4%) | 26(18.1%) | 0.401 | 8(16.7%) | 8(17.8%) | 0.887 |
| AKI | 26(38.8%) | 19(13.2%) | **＜0.001** | 20(41.7%) | 3(6.7%) | **＜0.001** |

* Some cases could not be evaluated due to the lack of reexamination of the pathogens. Bold font indicates data with significant differences.

**Table S3. Subgroup analysis of bloodstream infection**

| Treatment outcome | Before PSM | | | After PSM | | |
| --- | --- | --- | --- | --- | --- | --- |
|  | PMB  (N=26) | CAZ/AVI  (N=51) | p-value | PMB  (N=16) | CAZ/AVI  (N=23) | p-value |
| Clinical efficacy | 12(46.2%) | 39(76.5%) | **0.008** | 8(50.0%) | 18(78.3%) | 0.066 |
| 7-day microbiological clearance* | 3/22(13.6%) | 18/42(42.9%) | **0.037** | 0/12(0.0%) | 7/20(35.0%) | 0.061 |
| Microbiological clearance* | 6/18(33.3%) | 35/45(77.8%) | **0.001** | 1/11(9.1%) | 15/22(68.2%) | **0.005** |
| 30-day mortality | 7(26.9%) | 9(17.6%) | 0.343 | 5(31.3%) | 7(30.4%) | 0.957 |
| AKI | 8(30.8%) | 7(13.7%) | 0.074 | 6(37.5%) | 4(17.4%) | 0.297 |

* Some cases could not be evaluated due to the lack of reexamination of the pathogens. Bold font indicates data with significant differences.

**Table S4. Antibiotic susceptibility characteristic of 284 CRE strains**

| **Antibiotics and outcomes** | | **CAZ*** | **Polymyxins** | **Tigecycline** | **Amikacin** | **Sulfamethoxazole** |
| --- | --- | --- | --- | --- | --- | --- |
| *Klebsiella pneumoniae (276)* | Sensitive | 83（90.2%） | 236（85.5%） | 165（59.8%） | 52（18.8%） | 59（21.4%） |
|  | Moderate | N/A | 13（4.7%） | 63（22.8%） | N/A | N/A |
|  | Resistance | 9(9.8%) | 27（9.8%） | 48（17.4%） | 22（81.2%） | 217（78.6%） |
| *Escherichia coli (5)* | Sensitive | N/A | 5（100.0%） | 3（60.0%） | 2（40.0%） | N/A |
|  | Moderate | N/A | N/A | 2（40.0%） | N/A | N/A |
|  | Resistance | N/A | N/A | N/A | 3（60.0%） | 5（100.0%） |
| *Enterobacter cloacae (3)* | Sensitive | N/A | 3（100.0%） | 3（100.0%） | 3（100.0%） | 1（33.3%） |
|  | Moderate | N/A | N/A | N/A | N/A | N/A |
|  | Resistance | N/A | N/A | N/A | N/A | 2（66.7%） |

N/A, not applicable. All bacteria are resistant to meropenem. * Only 92 strains were carried out the antibiotic drug sensitivity test.

**Table S5.** **Univariate analysis of factors associated with clinical efficacy in CRKP-infected patients**

| **Demographics and clinical characteristics** | **Success (n=182)** | **Failure (n=94)** | **P-value** |
| --- | --- | --- | --- |
| **Demographic characteristics** |  |  |  |
| Age（years） | 58.5（46.0-68.0） | 66.0（47.8-73.3） | **0.003** |
| Gender (male) | 134（73.6%） | 58（61.7%） | **0.041** |
| Baseline creatinine (µmol/L) | 91.2（57.1-166.2） | 101.8（55.7-234.9） | 0.370 |
| Baseline CCR (mL/min) | 60.6（32.8-100.4） | 52.2（22.8-90.7） | **0.058** |
| RRT | 15（8.2%） | 12（12.8%） | 0.231 |
| Mechanical ventilation | 91（50.0%） | 72（76.6%） | **＜0.001** |
| Vasoactive drugs | 74（40.7%） | 58（61.7%） | **0.001** |
| ICU administration | 119（65.4%） | 73（77.7%） | **0.036** |
| Sepsis/Septic shock | 59（32.4%） | 52（55.3%） | **＜0.001** |
| Hospital stay (days) | 31.0（20.0-50.8） | 23.0（15.0-46.0） | **＜0.001** |
| APACHE II score | 24.1±8.5 | 27.0±10.6 | **＜0.001** |
| **Comorbidity** |  |  |  |
| Solid organ transplantation | 21（11.5%） | 3（3.2%） | **0.035** |
| Hypoproteinemia | 64（35.2%） | 41（43.6%） | 0.170 |
| Renal insufficiency | 31（17.0%） | 22（23.4%） | 0.203 |
| Diabetes mellitus | 55（30.2%） | 23（24.5%） | 0.315 |
| Digestive system Diseases | 90（49.5%） | 43（45.7%） | 0.559 |
| Cerebrovascular diseases | 66（36.3%） | 37（39.4%） | 0.614 |
| Cardiovascular diseases | 110（60.4%） | 51（54.3%） | 0.323 |
| Malignancy | 31（17.0%） | 14（14.9%） | 0.648 |
| **Infection sits** |  |  |  |
| Multi-site infection | 64（5.2%） | 27（28.7%） | 0.281 |
| Respiratory tract | 136（74.7%） | 75（79.8%） | 0.348 |
| Blood | 51（28.0%） | 26（27.7%） | 0.949 |
| Abdominal | 29（15.9%） | 12（12.8%） | 0.483 |
| Urinary tract | 38（20.9%） | 11（11.7%） | **0.059** |
| Central nervous system | 3（1.6%） | 1（1.1%） | 1.000 |
| Skin and soft tissue | 10（5.5%） | 3（3.2%） | 0.578 |
| **Pathogenic bacteria** |  |  |  |
| Only CRKP infection | 176（96.7%） | 92（97.9%） | 0.583 |
| CRKP + other CREs | 6（3.3%） | 2（2.1%） | 0.720 |
| **Antibiotic regimens** |  |  |  |
| Treatment duration (days) | 10.0（7.0-14.0） | 8.0（5.0-11.6） | **＜0.001** |
| Number of antibiotics | 1.0（0.0-1.0） | 1.0（0.0-1.0） | 0.851 |
| Preemptive therapy | 50（27.5%） | 28（29.8%） | 0.686 |
| +β-lactam | 28（15.4%） | 11（11.7%） | 0.405 |
| +Tigecycline | 35（19.2%） | 18（19.1%） | 0.987 |
| +Carbapenem | 55（30.2%） | 32（34.0%） | 0.517 |
| PMB-based regimens | 55（30.2%） | 43（45.7%） | **0.011** |
| CAZ/AVI-based regimens：Compared with PMB-based regimens | 127（69.8%） | 51（54.3%） |  |
| Monotherapy according to groups | 67（36.8%） | 33（35.1%） | 0.780 |

Abbreviations are the same as table 1. Bold font indicates data with significant differences.

**Table S6. Univariate analysis of factors associated with** **microbiological clearance in CRKP-infected patients**

| **Demographics and clinical characteristics** | **Total (N=216)** | **Clearance (N=138)** | **Failure (N=78)** | **P-value** |
| --- | --- | --- | --- | --- |
| **Demographic characteristics** |  |  |  |  |
| Age（years） | 58.0（46.0-68.0） | 58.0（45.0-69.0） | 58.5（47.0-68.0） | 0.844 |
| Gender (male) | 153（70.8%） | 102（73.9%） | 51（65.4%） | 0.185 |
| Baseline creatinine (µmol/L) | 88.7（55.6-169.1） | 108.8（59.8-197.5） | 74.8（51.8-143.8） | **0.031** |
| Baseline CCR (mL/min) | 65.9（31.6-102.4） | 56.3（27.5-98.5） | 74.5（38.8-120.7） | 0.114 |
| RRT | 21（9.7%） | 14（10.1%） | 7（9.0%） | 0.780 |
| Mechanical ventilation | 138（63.9%） | 89（64.5%） | 49（62.8%） | 0.806 |
| Vasoactive drugs | 106（49.1%） | 67（48.6%） | 39（50.0%） | 0.838 |
| ICU administration | 151（69.9%） | 100（72.5%） | 51（65.4%） | 0.276 |
| Sepsis/Septic shock | 84（38.9%） | 52（37.7%） | 32（41.0%） | 0.628 |
| Hospital stay (days) | 34.0（22.0-54.0） | 36.0（23.0-55.3） | 29.0（20.0-48.3） | 0.121 |
| APACHE II score | 23.5±7.8 | 23.8±8.3 | 23.0±6.7 | 0.509 |
| **Comorbidity** |  |  |  |  |
| Solid organ transplantation | 18（8.3%） | 17（12.3%） | 1（1.3%） | **0.010** |
| Hypoproteinemia | 81（37.5%） | 56（40.6%） | 25（32.1%） | 0.214 |
| Renal insufficiency | 40（18.5%） | 30（21.7%） | 10（12.8%） | 0.105 |
| Diabetes mellitus | 60（27.8%） | 45（32.6%） | 15（19.2%） | **0.035** |
| Digestive system diseases | 103（47.7%） | 65（47.1%） | 38（48.7%） | 0.819 |
| Cerebrovascular diseases | 78（36.1%） | 52（37.7%） | 26（33.3%） | 0.523 |
| Cardiovascular diseases | 127（58.8%） | 85（61.6%） | 42（53.8%） | 0.266 |
| Malignancy | 39（18.1%） | 28（20.3%） | 11（14.1%） | 0.256 |
| **Infection sits** |  |  |  |  |
| Multi-site infection | 84（38.9%） | 57（41.3%） | 27（34.6%） | 0.333 |
| Respiratory tract | 168（77.8%） | 110（79.7%） | 58（74.4%） | 0.364 |
| Blood | 63（29.2%） | 41（29.7%） | 22（28.2%） | 0.815 |
| Abdominal | 35（16.2%） | 19（13.8%） | 16（20.5%） | 0.196 |
| Urine tract | 45（20.8%） | 34（24.6%） | 11（14.1%） | 0.067 |
| Central nervous system | 3（1.4%） | 2（1.4%） | 1（1.3%） | 1.000 |
| Skin and soft tissue | 12（5.6%） | 10（7.2%） | 2（2.6%） | 0.257 |
| **Pathogenic bacteria** |  |  |  |  |
| Only CRKP infection | 210（97.2%） | 133（96.4%） | 77（98.7%） | 0.566 |
| CRKP+ other CREs | 6（2.8%） | 5（3.6%） | 1（1.3%） | 0.566 |
| **Antibiotic regimens** |  |  |  |  |
| Treatment duration (days) | 10.0（7.0-14.0） | 11.8（8.0-15.0） | 7.8（5.8-13.0） | **＜0.001** |
| Number of antibiotics | 1.0（0.0-1.0） | 1.0（0.0-1.0） | 1.0（1.0-1.0） | 0.093 |
| Preemptive treatment | 60（27.8%） | 42（30.4%） | 18（23.1%） | 0.246 |
| β-lactam | 33（15.3%） | 21（15.2%） | 12（15.4%） | 0.974 |
| Tigecycline | 47（21.8%） | 27（19.6%） | 20（25.6%） | 0.299 |
| Carbapenem | 67（31.0%） | 34（24.6%） | 33（42.3%） | **0.007** |
| CAZ/AVI-based regimens：Compared with PMB-based regimens | 146（67.6%） | 109（79.0%） | 37（47.4%） | **＜0.001** |
| Monotherapy according to groups | 72（33.3%） | 54（39.1%） | 18（23.1%） | **0.016** |

Abbreviations are the same as table 1. Bold font indicates data with significant differences.

**Table S7. Factors associated with 30-day all-cause mortality in patients with CRKP infection**

| **Demographics and clinical characteristics** | **Survivors (n=229)** | **Non-survivors (n=47)** | **HR（95%CI）** | **P-value** |
| --- | --- | --- | --- | --- |
| **Demographic characteristics** |  |  |  |  |
| Age（years） | 57.0（46.0-67.0） | 68.0（54.0-75.0） | 1.036（1.016-1.056） | **＜0.001** |
| Gender (male) | 162（70.7%） | 30（63.8%） | 0.756（0.417-1.371） | 0.358 |
| Baseline creatinine (µmol/L) | 89.6（56.7-162.5） | 104.7（59.6-196.1） | 1.001（0.999-1.003） | 0.457 |
| Baseline CCR (mL/min) | 66.6（33.3-103.3） | 45.8（28.3-83.5） | 0.994（0.988-1.000） | **0.045** |
| RRT | 20（8.7%） | 7（14.9%） | 1.623（0.727-3.624） | 0.237 |
| Mechanical ventilation | 125（54.6%） | 38（80.9%） | 3.237（1.565-6.697） | **0.002** |
| Vasoactive drugs | 97（42.4%） | 35（74.5%） | 3.767（1.953-7.263） | **＜0.001** |
| ICU administration | 156（68.1%） | 36（76.6%） | 1.403（0.714-2.756） | 0.326 |
| Sepsis/Septic shock | 80（34.9%） | 31（66.0%） | 3.266（1.786-5.974） | **＜0.001** |
| Hospital stay (days) | 33.0（21.0-53.0） | 24.0（15.0-39.0） | 0.985（0.972-0.998） | **0.023** |
| APACHE II score | 23.1±7.5 | 23.4（22.0-37.0） | 1.068（1.042-1.095） | **＜0.001** |
| **Comorbidity** |  |  |  |  |
| Solid organ transplantation | 24（10.5%） | 0（0.0%） | 0.042（0.001-2.829） | 0.140 |
| Hypoproteinemia | 89（38.9%） | 16（34.0%） | 0.899（0.492-1.644） | 0.730 |
| Renal insufficiency | 43（18.8%） | 10（21.3%） | 1.221（0.607-2.455） | 0.576 |
| Diabetes mellitus | 63（27.5%） | 15（31.9%） | 1.164（0.631-2.150） | 0.627 |
| Digestive system Diseases | 109（47.6%） | 24（51.1%） | 1.106（0.624-1.959） | 0.731 |
| Cerebrovascular diseases | 86（37.6%） | 17（36.2%） | 0.942（0.520-1.708） | 0.844 |
| Cardiovascular diseases | 130（56.8%） | 31（66.0%） | 1.482（0.811-2.710） | 0.201 |
| Malignancy | 38（16.6%） | 7（14.9%） | 1.006（0.451-2.247） | 0.988 |
| **Infection sits** |  |  |  |  |
| Multi-site infection | 76（33.2%） | 15（31.9%） | 0.924（0.500-1.706） | 0.800 |
| Respiratory tract | 176（76.9%） | 35（74.5%） | 0.881（0.457-1.697） | 0.704 |
| Blood | 61（26.6%） | 16（34.0%） | 1.364（0.746-2.495） | 0.313 |
| Abdominal | 33（14.4%） | 8（17.0%） | 1.180（0.552-2.526） | 0.669 |
| Urinary tract | 44（19.2%） | 5（10.6%） | 0.538（0.213-1.360） | 0.190 |
| Central nervous system | 4（1.7%） | 0（0.0%） | - | - |
| Skin and soft tissue | 11（4.8%） | 2（4.3%） | 0.827（0.201-3.411） | 0.793 |
| **Pathogenic bacteria** |  |  |  |  |
| Only CRKP infection | 221（96.5%） | 47（100.0%） | **-** | **-** |
| CRKP+ other CREs | 8（3.5%） | 0（0.0%） | - | - |
| **Antibiotic regimens** |  |  |  |  |
| Treatment duration (days) | 10.0（7.0-14.0） | 7.0（5.0-11.0） | 0.891（0.833-0.953） | **0.001** |
| Number of antibiotics | 1.0（0.0-1.0） | 1.0（0.0-1.0） | 0.992（0.713-1.380） | 0.960 |
| Preemptive therapy | 63（27.5%） | 15（31.9%） | 1.123（0.608-2.073） | 0.712 |
| β-lactam | 33（14.4%） | 6（12.8%） | 0.852（0.362-2.008） | 0.715 |
| Tigecycline | 43（18.8%） | 10（21.3%） | 1.157（0.575-2.326） | 0.683 |
| CAZ/AVI-based regimens | 147（64.2%） | 31（66.0%） | 1.132（0.619-2.070） | 0.687 |
| Monotherapy according to groups | 84（36.7%） | 16（34.0%） | 0.947（0.518-1.731） | 0.859 |

Abbreviations are the same as table 1. Bold font indicates data with significant differences.

**Table S8. Univariate analysis of factors associated with AKI in CRKP-infected patients**

| **Demographics and clinical characteristics** | **Non-AKI (n=219)** | **AKI (n=57)** | **P-value** |
| --- | --- | --- | --- |
| **Demographic characteristics** |  |  |  |
| Age（years） | 58.0（46.0-69.0） | 60.0（47.0-68.0） | 0.795 |
| Gender (male) | 151（68.9%） | 41（71.9%） | 0.663 |
| Baseline creatinine (µmol/L) | 92.3（59.8-172.8） | 86.4（52.9-132.0） | 0.250 |
| Baseline CCR (mL/min) | 62.0（29.0-96.1） | 59.2（39.5-120.9） | 0.273 |
| RRT | 21（9.6%） | 6（10.5%） | 0.832 |
| Mechanical ventilation | 129（58.9%） | 34（20.9%） | 0.919 |
| Vasoactive drugs | 103（47.0%） | 29（50.9%） | 0.605 |
| ICU administration | 153（69.9%） | 39（68.4%） | 0.833 |
| Sepsis/Septic shock | 92（42.0%） | 19（33.3%） | 0.234 |
| Hospital stay (days) | 31.0（19.0-49.0） | 28.0（22.0-61.5） | 0.391 |
| APACHE II score | 24.0±8.8 | 24.8±7.4 | 0.493 |
| **Comorbidity** |  |  |  |
| Solid organ transplantation | 18（8.2%） | 6（10.5%） | 0.582 |
| Hypoproteinemia | 84（38.4%） | 21（36.8%） | 0.834 |
| Renal insufficiency | 42（19.2%） | 11（19.3%） | 0.984 |
| Diabetes mellitus | 66（30.1%） | 12（21.1%） | 0.175 |
| Digestive system Diseases | 105（47.9%） | 28（49.1%） | 0.874 |
| Cerebrovascular diseases | 79（36.1%） | 24（42.1%） | 0.402 |
| Cardiovascular diseases | 124（56.6%） | 37（64.9%） | 0.258 |
| Malignancy | 37（16.9%） | 8（14.0%） | 0.603 |
| **Infection sits** |  |  |  |
| Multi-site infection | 76（34.7%） | 15（26.3%） | 0.230 |
| Respiratory tract | 166（75.8%） | 45（78.9%） | 0.618 |
| Blood | 62（28.3%） | 15（26.3%） | 0.765 |
| Abdominal | 34（15.5%） | 7（12.3%） | 0.540 |
| Urinary tract | 42（19.2%） | 7（12.3%） | 0.225 |
| Central nervous system | 4（1.8%） | 0（0.0%） | - |
| Skin and soft tissue | 12（5.5%） | 1（1.8%） | 0.406 |
| **Pathogenic bacteria** |  |  |  |
| Only CRKP infection | 214（97.9%） | 54（94.7%） | 0.452 |
| CRKP+ other CREs | 5（2.3%） | 3（5.3%） | 0.452 |
| **Antibiotic regimens** |  |  |  |
| Treatment duration (days) | 10.0（6.5-14.0） | 10.0（7.0-14.0） | 0.829 |
| Number of antibiotics | 1.0（0.0-1.0） | 1.0（0.0-1.0） | 0.253 |
| Preemptive therapy | 59（26.9%） | 19（33.3%） | 0.340 |
| +SMZ | 6（2.7%） | 4（7.0%） | 0.254 |
| + Quinolones | 16（7.3%） | 5（8.8%） | 0.710 |
| + Aminoglycosides | 14（6.4%） | 2（3.5%） | 0.407 |
| +β-lactam | 27（12.3%） | 12（21.1%） | 0.092 |
| +Tigecycline | 40（18.3%） | 13（22.8%） | 0.438 |
| +Carbapenem | 67（30.6%） | 20（35.1%） | 0.515 |
| PMB-based regimens | 65（29.7%） | 33（57.9%） | **＜0.001** |
| CAZ/AVI-based regimens | 154（70.3%） | 24（42.1%） |  |
| Monotherapy according to groups | 84（38.4%） | 16（28.1%） | 0.150 |

Abbreviations are the same as table 1. Bold font indicates data with significant differences.
